# Computational epitope-based vaccine design with bioinformatics approach; a review

**DOI:** 10.1016/j.heliyon.2025.e41714

**Published:** 2025-01-04

**Authors:** Esmaeil Roohparvar Basmenj, Susan Radman Pajhouh, Afsane Ebrahimi Fallah, Rafe naijian, Elmira Rahimi, Hossein Atighy, Shadan Ghiabi, Shamim Ghiabi

**Affiliations:** a Biophysics Department, Faculty of Biological Sciences, Tarbiat Modares University, Tehran, Iran; b Faculty of Agricultural Sciences, Shahed University, Tehran, Iran; c Faculty of Life Sciences and Biotechnology, Shahid Beheshti University, Tehran, Iran; d Student research committee, faculty of pharmacy, Mazandaran University of Medical Sciences, Sari, Iran; e Department of Biology, Central Tehran Branch, Islamic Azad University, Tehran, Iran; f School of Pharmacy, Centro Escolar University, Manila, Philippines; g Faculty of Veterinary Medicine, Science and Research Branch, Islamic Azad University, Tehran, Iran; h Tehran Azad University of Medical Sciences, Faculty of Pharmaceutical Sciences, Iran

**Keywords:** Vaccine design, Reverse vaccinology, Multi epitope vaccine, Bioinformatics, Immunoinformatics

## Abstract

The significance of vaccine development has gained heightened importance in light of the *COVID-19* pandemic. In such critical circumstances, global citizens anticipate researchers in this field to swiftly identify a vaccine candidate to combat the pandemic's root cause. It is widely recognized that the vaccine design process is traditionally both time-consuming and costly. However, a specialized subfield within bioinformatics, known as "multi-epitope vaccine design" or "reverse vaccinology," has significantly decreased the time and costs of the vaccine design process. The methodology reverses itself in this subfield and finds a potential vaccine candidate by analyzing the pathogen's genome. Leveraging the tools available in this domain, we strive to pinpoint the most suitable antigen for crafting a vaccine against our target. Once the optimal antigen is identified, the next step involves uncovering epitopes within this antigen. The immune system recognizes particular areas of an antigen as epitopes. By characterizing these crucial segments, we gain the opportunity to design a vaccine centered around these epitopes. Subsequently, after identifying and assembling the vital epitopes with the assistance of linkers and adjuvants, our vaccine candidate can be formulated. Finally, employing computational techniques, we can thoroughly evaluate the designed vaccine. This review article comprehensively covers the entire multi-epitope vaccine development process, starting from obtaining the pathogen's genome to identifying the relevant vaccine candidate and concluding with an evaluation. Furthermore, we will delve into the essential tools needed at each stage, comparing and introducing them.

## Graphical abstract

**Figure:**
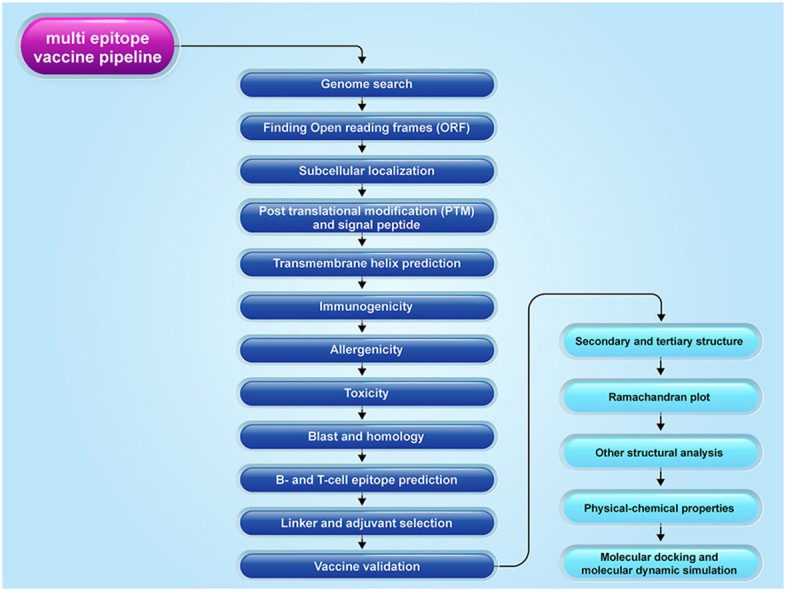

## 1 Introduction

Different approaches for designing vaccines are available, but they confront significant obstacles because of the immune system's complexity and the body's complex systems. The primary challenge inherent in traditional methods is the substantial time and financial resources required to dissect intricate bodily systems and processes. In the realm of vaccine design, however, computational and bioinformatics techniques have emerged as valuable tools. During the computational vaccine candidate design process, upon identifying the optimal antigen The epitopes that can trigger the highest immune responses can be investigated.

Antigens, in this context, are substances that, upon entry into the body, trigger immune reactions leading to the production of antibodies. Epitopes, on the other hand, represent specific portions of antigens recognized by antibodies, B cells, or T cells. Through the integration of computational techniques and the alignment of the three key components—antigens, antibodies, and epitopes—The efficiency of immune system simulation increases.

The incorporation of computational tools offers a promising avenue for expediting vaccine development and enhancing effectiveness, especially when it comes to treating newly discovered infectious illnesses and enhancing immunogenicity. To embark on this journey, it is essential to first gain a comprehensive understanding of the immune system and grasp the fundamental principles of its operation.

This article will explore the various levels of the immune system and elucidate the fundamental biological knowledge necessary for crafting vaccines. Subsequently, it will provide a detailed examination of all the steps required for designing a multi-epitope vaccine.

## 2 Immunity system and immunogenicity

Edward Jenner made the first attempt to immunize the body against the disease in 1796, which showed that humans could be protected from smallpox by inoculating the material collected from the lesion of a cowpox-infected human [1]. This was a breakthrough in medical history and the beginning of the practice known as vaccination. To stimulate the immune system against the targeted pathogen, vaccines are biological preparations. Injuries should not be severe as a result of the vaccination. Still, it ought to increase the vaccinated host's immune system so that the body's defenses can identify the invader and eliminate it if an infection later occurs [2]. The response to a foreign substance is called immunity, and the science of immunology is the study of cellular and molecular events upon encountering a foreign substance. The term "immunity" was developed to characterize people who had recovered from specific infectious diseases and were protected from re-exposure to the same diseases.

The cells and molecules that cause immunity, and the body's response to components of microbes, macromolecules such as proteins, polysaccharides, and small chemicals are called the immune response [3].

The mechanism related to the body's immune response is the reason for the effectiveness of the vaccination method. This mechanism is based on identifying features of the structure of pathogens so that they can distinguish them from the host cells and quickly give an appropriate defensive response after encountering them [4]. The ability to distinguish host cells from pathogens to destroy them without damaging internal tissues is essential for the immune system.

### 2.1 The innate and adaptive immune system

There are two types of immune systems: innate and adaptive [3]. where all multicellular organisms have innate immunity and include non-specific defense mechanisms, on the contrary, adaptive immunity is dedicated to higher-order creatures like vertebrates.

Lymphocyte cells, including B and T cells, are the main compartments of the adaptive immune system and generate humoral and cellular immunity, respectively. However, their mechanisms of action differ significantly, they produce long-lived cells that can remember previous infections and respond even more intensely to subsequent encounters with the previous pathogens, even decades later.

The genetic recombination that occurs during the maturation of lymphocytes results in the production of millions of distinct lymphocyte clones in terms of their receptor classes, giving them specificity for antigens critical to the proper functioning of the adaptive immune system [5,6].

#### 2.1.1 B lymphocytes

Bone marrow cells generate and develop B lymphocytes and can trigger immune responses [7]. In a simple definition, B lymphocytes are a population of cells expressing immunoglobulins or antibodies with various cell surface receptors capable of recognizing antigen epitopes. B cells were not initially identified within cells themselves but rather through the discovery of antibodies [8]. It is important to note that in early 1948, plasma cells were introduced as a source of antibody production [9,10].

B cells were discovered and characterized in the mid-1960s and early 1970s through studies involving model laboratory animals and clinical evaluations of patients with immunodeficiency diseases [11].

B cells are classified into four types: memory, naive, plasma, and transitional [11]. Transitional B cells are the first cells to migrate to the spleen, lymph nodes, and peripheral blood after developing from precursors in the bone marrow. Immature B cells are derived from these cells.

Transitional B cells act as a bridge between immature and mature B cells. Naive B cells represent the next stage beyond transitional B cells. When a transitional B cell is located in the spleen, tonsils, or lymph nodes, it is referred to as a naive B cell. When naive B cells are exposed to antigen-presenting cells (APCs) that match their receptors, they mature and can then become plasma or memory B cells that secrete antibodies specific to the originally bound antigen.

The memory B cell is a significant B cell subtype that is necessary for the body to develop long-term immunity. After an infection has subsided, If the host is exposed to the same antigen again, it remains in the bloodstream and can quickly become active again. Large cells with a large endoplasmic reticulum (ER) that synthesizes and transports proteins enable plasma B cells, also referred to as effector B cells, to produce a substantial quantity of antigen-specific antibodies. When signal chemicals occur, they respond by continuing to produce antibodies to fight infections until they are under control or eliminated. This activity of the B cells is called humoral immunity [12].

The most crucial aspect of B cell immunity progression is that the majority of B cells die within a few days, and only a few B cells persist in the bone marrow or continue to circulate in the blood for months or even years [13]. B cells collaborate with other ingredients of the immune system to prevent the entry of pathogens and foreign substances.

#### 2.1.2 T lymphocytes

T lymphocytes are the key elements of the vertebrate immune system, in charge of eliciting a highly specific and long-lasting immune response against invaders of the pathogen. T lymphocytes comprise a set of cell clones specialized to deal with a variety of environmental antigens and each can recognize a specific antigen [14]. They mature in the thymus and are released as naive T cells into the bloodstream. Naive T cells search for antigen-presenting cells (APC) and can mature in response to TCR, BCR, or cytokine signals it receive upon encountering a recognized APC. If all three signals are received, the cell matures into an effector cell. Effector T cells have short lifespans and carry out immune response functions.

Four main types of T cells can be cytotoxic, helper, regulatory, or memory [15].

• CD8 cells also called cytotoxic T cells use apoptosis to kill toxic/target cells and remove rapidly infected cells, bacteria, and tumor fragments.
• CD4 cells also called T helper cells, like cytotoxic cells, perform a wider range of functions and are required for most adaptive immune responses.
• Regulatory T cells stop an autoimmune response once the threat has been eliminated, preventing helper T cells from taking up space or accidently attacking healthy cells.
• Memory T cells are long-living lymphocytes that can respond to antigens when they are reintroduced, helping the body to build immunological tolerance [16].

### 2.2 Major histocompatibility complex (MHC)

The Major Histocompatibility Complex (MHC) is a set of genes that produce proteins that exist on the cell surfaces and help the immune system in identifying foreign components [17]. Another name for it is the human leukocyte antigen (HLA) system and is found in all higher vertebrates.

Class I and class II MHC protein molecules are the two main subtypes. Nearly every cell in an organism has class I MHC molecules, whereas class II molecules are only found in immune system cells like macrophages and lymphocytes [18]. In humans, these molecules are encoded by several genes clustered on chromosome 6, each with an unusually large number of alleles.

#### 2.2.1 MHC class 1

The major histocompatibility complex (MHC) class I antigen presentation pathway plays a crucial role in alerting the immune system to cells infected by viruses. All nucleated body cells express MHC class I molecules on their cell surface, which are made up of peptide fragments that are derived from intracellular proteins [19]. These peptides are transported to the cytosol via an antigen-processing-associated transporter (TAP).

MHC class I molecules have deep binding pockets with specific physicochemical preferences in their binding cleft [20], such as hydrophobic and hydrophobic residues at the carboxyl end of peptides. After binding of peptides to MHC1 molecules, the complex is released from the endoplasmic reticulum and transferred to the cell surface, activating CD8^+^ cytotoxic T lymphocytes (CTLs) [21,22] after processing the antigen peptides in the peptide-MHCI complex [6].

#### 2.2.2 MHC class 2

MHC Class II proteins are mainly expressed on antigen-presenting cells, such as dendritic cells, macrophages, and B cells, although MHC Class I proteins are present on the surface of nearly all nucleated cells in the body. Major Histocompatibility Complex (MHC) class II molecules mainly bind and present exogenous protein fragments to CD4^+^ T-helper cells, which then regulate B cell development in antibody-producing B-cell blasts [19].

The binding groove on MHC Class II molecules is open-ended and can hold peptides of different lengths. T helper cells contribute to antibody-induced immunity, inflammatory responses, defense against extracellular bacteria, and cell-mediated immunity against intracellular pathogens [23].

### 2.3 B & T cell epitope

**Fig. 1:**
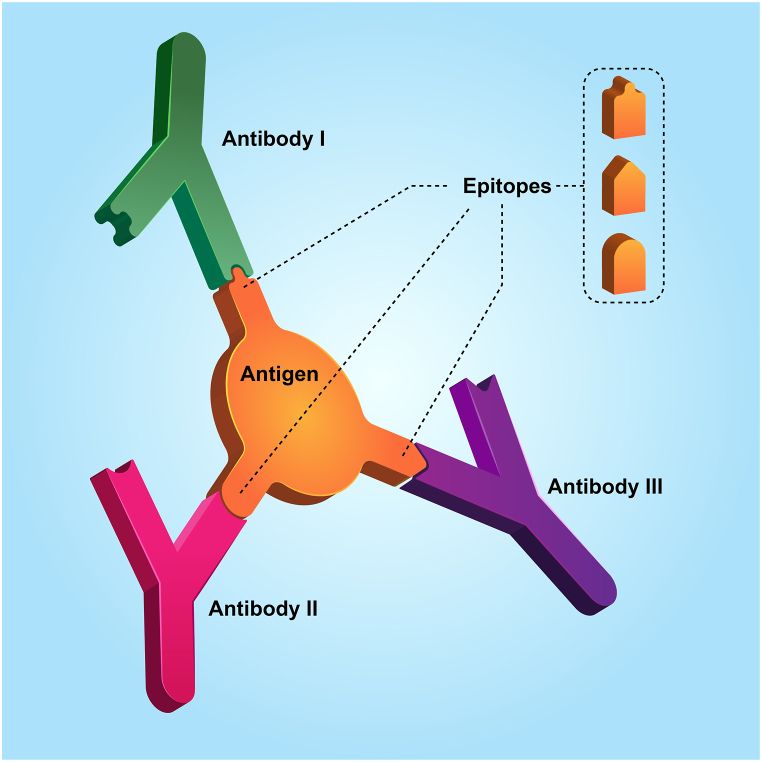
The specificity of epitope-antibody binding.

Foreign substances recognized by lymphocytes and antibodies are called antigens. The ability to recognize and specifically link an antigen with T&B cell receptors is related to limited parts of the antigen, called antigenic indicators or epitopes. Specific receptors recognize these epitopes on the surface of B and T cells [39]. The specificity of epitope-antibody binding is crucial to note, as each antibody recognizes its unique and specific epitope in a complementary manner. Illustrated in Fig. 1, it is evident that antibody I is incapable of recognizing the epitope acknowledged by antibody II. This distinct binding specificity ensures that antibodies selectively interact with their designated epitopes and not with those recognized by other antibodies.

**Fig. 2:**
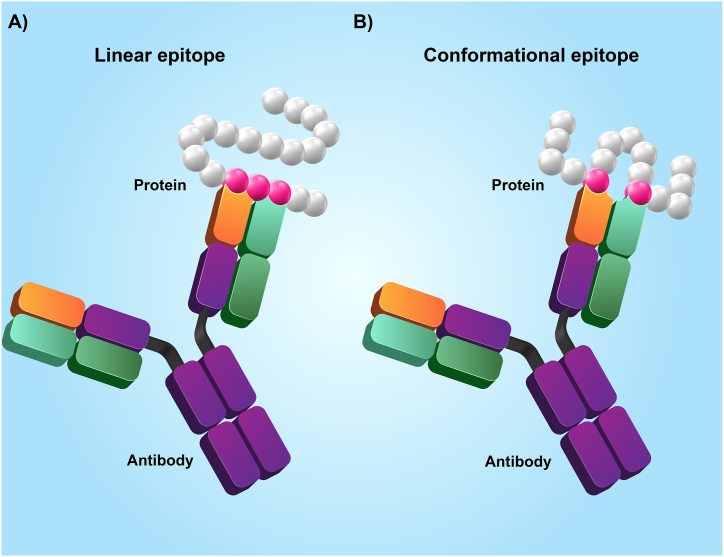
A: Linear epitope. B: Conformational epitope.

The host's immune system identifies the epitope and activates the body's protection against an invasive pathogen. Only when the epitope binds to its complementary structure immune response is activated. Epitope types include Conformational (discontinuous) and Linear (continuous), as depicted in Fig. 2.

The indices at the antigen molecule's corners are called conformational epitopes recognized by B-lymphocyte cells and antibodies [40]. Conformational epitopes featuring discontinuities emerge through the process of protein folding. These epitopes materialize when non-adjacent segments of peptide chains come together.

Labels that include individual residues of amino acids within the protein sequence and are on the flat surface of the molecule are called linear epitopes. Linear epitopes are recognized by B and T lymphocytes and MHC cells [41,42].

T- and B-cells provide an immune response based on memory specific to a particular infection. A B-cell epitope is the area of the antigen that immunoglobulins or antibodies bind to. T-cell epitopes are found on the surface of antigen-presenting cells and are linked to major histocompatibility complex components.

#### 2.3.1 Epitope prediction

There are many reasons for the prediction of epitopes in antigens; for example, Epitopes provide important information for the diagnosis, treatment, and prevention of disease, realizing pathogenicity mechanisms, immune system surveillance, developing diagnostic methods, and designing epitope-based vaccines [24]. Epitope identification and discovery have been in the spotlight for years, particularly in the design, synthesis, and development of artificial vaccine platforms.

In the past, the development of vaccines was entirely dependent on immunological and biochemical experiments, such as.

• phage display library
• overlapping peptides
• ELISA
• NMR
• Immuno-flurorescence
• Radioimmunoassay
• Western-blotting
• Immunohistochemistry
• X-ray crystallography studies of antibody/antigen structure
• attenuation of the wild-type pathogens by random mutations and serial passages.

These experiments are very expensive and time-consuming [25]. However, epitope mapping, the process of identifying all possible epitopes, has significantly helped reduce both time and cost. This progress in research supports vaccine design by enabling the prediction of specific epitopes. Instead of relying on the entire antigen, researchers can now concentrate on utilizing these predicted epitopes for the development of their vaccine candidates [5].

### 2.4 Reverse vaccinology

For years, researchers have been working to develop vaccines for various diseases, testing different solutions and technologies. Now, there are multiple approaches available for designing vaccines. However, traditional methods and common laboratory and test procedures can be time-consuming and costly, making the research process challenging [26].

In the vaccine design process, it is crucial to identify all the proteins encoded by the pathogen. Imagine a scenario where we can pinpoint the regions in the pathogen's genome that have the potential to be converted into proteins. In this case, it is evident that the key antigens of the pathogen, which can trigger an immune response upon entering the body, will be among the identified proteins. This represents a significant advancement in vaccine research. This approach involves working backward from the pathogen's genome, systematically identifying all potentially coded proteins in the first step and then using various filters to narrow down and identify the most effective antigens [27].

This approach is known as reverse vaccinology, which begins with the genome rather than the microorganism, taking the opposite direction. The reverse vaccinology method has the potential to address major obstacles in vaccine development, such as non-cultivable microorganisms and antigens that cannot be expressed in in-vitro conditions. Reverse vaccinology has been developed as a promising strategy for delivering therapeutic and preventive effects on pathogen-specific immunity. This approach also helps to decrease the cost and time needed for research [28].

The reverse vaccinology approach involves five key steps.

1. Identifying all proteins encoded by the pathogens.
2. Selecting the best antigens from among these proteins.
3. Using in silico epitope mapping to predict immunogenic regions
4. Engineering the immunogenic structure.
5. Evaluating the vaccine's efficiency.

The vaccine produced using the reverse vaccinology approach is called an epitope-based vaccine. This article aims to describe the step-by-step process of epitope-based vaccine design by introducing the best databases and software tools for each step.

## 3 Multi-epitope vaccine pipeline

**Fig. 3:**
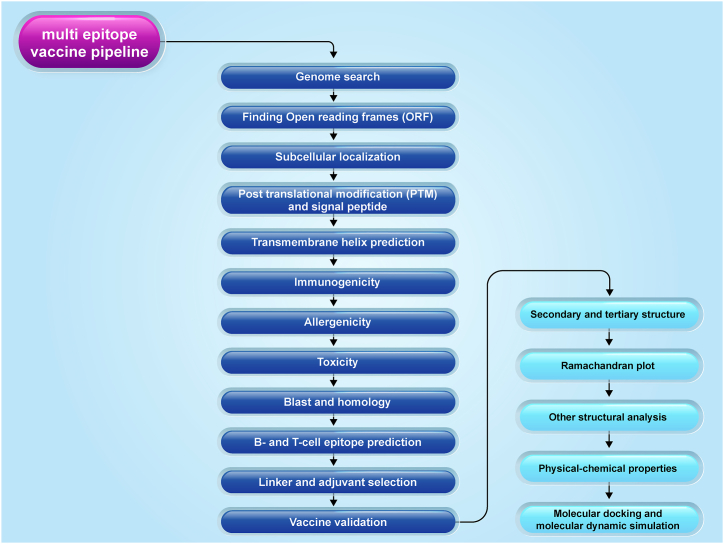
The methodology for designing multi-epitope vaccines using bioinformatics approaches.

The development of a multi-epitope vaccine begins with the identification of the pathogen's genome. From there, we select the most suitable antigens and epitopes to create an effective vaccine. In this section, we will outline each stage of this process and discuss the essential tools available for this purpose. The methodology for designing multi-epitope vaccines using bioinformatics approaches is depicted in Fig. 3.

### 3.1 Genome search

The first stage in creating epitope-based vaccinations is to download the target pathogen's DNA. Numerous servers are available for researchers to obtain genomes, and here, we aim to introduce the most important ones.

#### 3.1.1 NCBI

The National Center for Biotechnology Information (NCBI), is a center that collects data related to genome and biomedical information and makes it available to researchers through NCBI's website [29]. This server has many parts, one of which is the Genome. The NCBI genome database contains sequence information and maps of all the genomes of nearly a thousand species or breeds. The Genome section contains genomes whose sequence is complete and those whose sequencing is in progress. All three major kingdoms of life (bacteria, archaea, and eukaryotes) are represented, plus many viruses, phages, viroids, plasmids, and organelles.

#### 3.1.2 Ensembl

Ensembl is a bioinformatics project to organize sequences of large genomes and biological data specifically for vertebrates [30]. It serves as a framework for combining any biological data that can be mapped onto features found in the genome. Ensembl is a portable, open-source software system for managing genomes and an interactive website and collection of flat files. The code is freely available, and all data are given without limitations.

#### 3.1.3 EBI

The European Bioinformatics Institute (EBI) maintains a comprehensive collection of primary nucleotide sequences known as the European Molecular Biology Laboratory (EMBL) Nucleotide Sequence Database [31]. The world's most extensive collection of freely accessible and current molecular data resources is kept up to date by the European Bioinformatics Institute (EMBL-EBI) of EMBL. The scientific services provided by EMBL include more than 40 bioinformatics and data resources, as well as 20 services for experiments in chemical biology, imaging, proteomics, metabolomics, genomics, in vivo gene editing, and structural biology. Researchers can view each of the 23 chromosomes of the human genome at any scale, from a whole chromosome to a single nucleotide, due to the UCSC Genome Browser, an online tool that serves as a multi-powered microscope [32]. popular web-based tool for rapidly displaying a desired region of a genome at any scale is the genome browser, which also displays a set of aligned annotation "tracks" for the region. In addition to the servers we introduced, there are other public genome download databases in various domains, as listed in Table 1.

**Table 1:** list of genome download databases.

Database	URL	Description	Ref
**NCBI**	https://www.ncbi.nlm.nih.gov/	center that collects data related to genome and biomedical information through the NCBI website	[33]
**Ensembl**	https://asia.ensembl.org/index.html	Ensembl provides reference datasets and analysis tools that enable genomics studies; based on EMBL-EBI and freely available.	[30]
**EMBL**	https://www.embl.org/	collection of primary nucleotide sequences.	[34]
**Gramene**	https://www.gramene.org/	unique database for genetic and genomic studies on plant genomes	[35]
**UCSC**	https://genome.ucsc.edu/	Gene predictions, mRNA and expressed sequence tag alignments, expression and regulatory information, and pairwise and multiple-species comparative genomics data are all displayed.	[36]
**DDBJ**	https://www.ddbj.nig.ac.jp/index-e.html	DNA sequences are gathered for a biological database. It can be found in Japan at the National Institute of Genetics (NIG).	[37]
**KEGG**	https://www.genome.jp/kegg/	a database for the systematic study of gene functions that connect genomic data with higher level functional data	[38]
**MGD**	http://www.informatics.jax.org	The genetic and genome resource for laboratory mice in the community model organism	[39]
**ZFIN**	https://zfin.org/	the genetic and genomic database for the model organism zebrafish (Danio rerio).	[40]
**dictyBase**	http://dictybase.or	database of genetic and genomic data for the Dictyostelium discoideum as a model organism	[41]
**IGSR**	https://www.internationalgenome.org	A worldwide collection of genome variation incorporating the 1000 Genomes Project data	[42]
**RGD**	https://rgd.mcw.edu	database based on genomic and genetic information about rats, a model organism	[43]
**TAIR**	https://www.arabidopsis.org	genetic and molecular biology database for Arabidopsis thaliana, a model plant	[44]

### 3.2 Finding open reading frames (ORF)

In the process of designing multi-epitope vaccines, the initial step is to identify antigens with high immunogenic potential. After identifying all antigens, a systematic screening approach with restrictions and filters is employed to choose the best antigens with high immunogenic properties. Subsequently, potential epitopes on this selected antigen are identified to construct the vaccine.

The important note in this regard is that, on the genome of the pathogen, there are some regions with the ability to translate into proteins. In other words, all the mentioned antigens come from this crucial region on the genome. This region is called ORF. So, if we identify all the ORFs on the genome, we have the opportunity to access all potential antigens. Typically, an ORF starts with a start codon (ATG) as its initiation point and concludes with a stop codon (TAA, TAG, or TGA). In other words, an ORF is defined as a subsequence that commences with the start codon and terminates with an end codon. It's important to note that in some species, there may be variations in the codon table, and their start or stop codons, or the entire codon table, may differ.

The process of identifying ORFs has become more streamlined, thanks to advanced bioinformatics tools and servers. These servers can analyze the genome of the pathogen, predict all potential ORFs, and provide valuable insights into potential antigens. Some noteworthy ORF finder servers include the following.

#### 3.2.1 ORF-finder

The ORFfinder server at https://www.ncbi.nlm.nih.gov/orffinder/is made to search for open reading frames, or ORFs, in the input DNA sequence [45]. This program provides each ORF's region and the translation of its protein.

RNA or DNA sequences can be used to find ORF regions. Also, in this server, the end and start codons, the length of the ORF, and its genetic coding can be specified for a more detailed search. In the end, you can use the SMART-BLAST or BLAST tool to compare and align the sequence of ORFs with the sequence of known proteins to determine the function of the identified possible protein. The web version of ORF Finder is limited to a subset of input sequences that are longer than 50,000 nucleotides. A standalone version, which does not limit the length of the input sequence, is available for Linux X64. Enter the desired sequence as the accession number and gi or nucleotide sequence in FASTA format in the text area**.**

#### 3.2.2 Sequence Manipulation Suite

The Sequence Manipulation Suite (https://sites.ualberta.ca/∼stothard/javascript/orf_find.html) comprises JavaScript programs designed for generating, formatting, and analyzing short DNA and protein sequences. Widely used by molecular biologists for teaching and testing programs and algorithms.

Among these apps is ORF Finder. Open reading frames, or ORFs, are found in an input DNA sequence using ORF Finder. This program returns the region of each ORF and its protein translation. Find potential protein-coding regions in newly sequenced DNA using ORF Finder. You can use the Sequence Manipulation Suite online, or you can download a copy of the program and save it to your computer for offline use.

#### 3.2.3 starORF

StarORF, available at http://star.mit.edu/orf/index.html, facilitates the identification of proteins encoded by DNA sequences. Using StarORF. After a DNA sequence is transcribed to RNA, all possible ORFs are identified on it [46].

#### 3.2.4 Expasy translate

The SIB Swiss Institute of Bioinformatics' bioinformatics resource portal is called Expasy, and it can be found at https://web.expasy.org. "Translate," one of its features, makes it easier to translate a nucleotide (DNA/RNA) sequence into the corresponding protein sequence [47].

#### 3.2.5 Critical analysis of open reading frame (ORF) detection methods

Among the tools discussed in this section, **OrfFinder** is the most commonly used. It provides various codon tables and is known for its high speed. However, its main limitation is that it can only process genomes up to 50,000 bases in length. To work around this, users must divide their sequence into 50,000-base sections and run the server for each part. Additionally, it supports running the server using either an accession number or a GI number.

In contrast, **OrfStar** ignores missing bases that may occur during the sequencing phase. We do not recommend this tool, as ignoring missing bases can lead to shifts in codon reading frames, resulting in inaccurate predictions.

Regarding the **Sequence Manipulation Suite** server, it has an input limit of 100,000,000 characters, but it does not allow users to run the server with an accession number or GI. Moreover, this server does not offer an option to ignore nested ORFs.

One advantage of the **ExPASy Translate** server is that users can specify the output format. However, it does not allow for further customization of the algorithmic procedure.

#### 3.2.6 Future research directions

For future research, we suggest developing a server that includes the following features.

• Support for all codon tables.
• No input character limitations.
• Ability to run the server with an accession number or GI.
• Option to input sequences via file upload.
• Capability to process multiple genomes simultaneously and return ORFs for each genome in separate files.
• Identification of output format.
• User-configurable settings, such as:
  o Minimum ORF length.
  o Start and stop codon selection.
  o Option to ignore or not ignore nested ORFs.
  o Option to specify whether to find ORFs on the direct strand or reverse strand.

### 3.3 Subcellular localization

In this phase, our focus should be directed towards proteins that are exposed to the immune system. Indeed, for a protein to serve as a viable candidate for multi-epitope vaccine research, it must be recognized by the immune system as an antigen, prompting the production of antibodies against it. Proteins that remain unexposed to the immune system are unsuitable for consideration in this context.

This leads us to the crucial topic of subcellular localization. Armed with the protein sequence, we gain insights into the specific cellular compartments to which a protein belongs. However, the challenge lies in envisioning the diverse locations a protein may occupy. Consider, for instance, the following categories representing potential protein locations.

1. Cytoplasmic
2. Cytoplasmic Membrane
3. Periplasmic
4. Outer Membrane
5. Extracellular
6. And …

Among these, extracellular and outer membrane proteins emerge as particularly promising targets for multi-epitope research. The rationale behind this preference is rooted in the fact that these proteins exist outside the cell, rendering them readily visible to the immune system. It is important to acknowledge that, depending on the specifics of your research, intracellular locations may be chosen as targets in certain scenarios. Nonetheless, for multi-epitope exploration, extracellular and outer membrane proteins are notably advantageous. At this juncture, employing the servers outlined in Table 2 is instrumental in identifying proteins that are indeed exposed to the immune system.

**Table 2:** List of protein subcellular localization prediction tools.

Server	URL	Description	Ref
**UniProtKB**	https://www.uniprot.org/	A comprehensive database of protein sequences that have been carefully selected by biologists is called the UniProt-Knowledge Base (UniProtKB). It establishes cross-links with UniProt Reference Clusters (UniRef). Protein sequence clusters with 100 %, 90 %, or 50 % identity are defined by the UniRef databases.	[48]
**LOCATE**	http://locate.imb.uq.edu.au/	The web-based database LOCATE contains information about the membrane organization and subcellular localization of mouse and human proteins.	[49]
**PSORTb**	http://www.psort.org/psortb	PSORTb remains the most accurate predictor of bacterial subcellular protein localization (SCL).	[50]
**Eslpred**	http://www.imtech.res.in/raghava/eslpred/	Predicts the subcellular localization of eukaryotic proteins based on amino acid composition, dipeptide composition, and physicochemical properties.	[51]
**Cell-PLoc**	http://chou.med.harvard.edu/bioinf/Cell-PLoc	The Euk-mPLoc, Hum-mPLoc, Plant-PLoc, Gpos-PLoc, Gneg-PLoc, and Virus-PLoc Web server packages, which are specific to eukaryotic, human, plant, Gram-positive, Gram-negative, and viral proteins, are all included in the recently developed Cell-PLoc Web server package.	[52]
**locDB**	https://www.rostlab.org/	The database LocDB is a database of experimental annotations on the subcellular localization of proteins in humans (Homo sapiens) and Arabidopsis thaliana, carefully selected by experts. For every protein with experimental data, the database includes subcellular localization predictions using a variety of cutting-edge prediction techniques.	[53]
**SCLpred-EMS**	http://distilldeep.ucd.ie/SCLpred2/.	A group of deep N-to-1 convolutional neural networks is used in SCLpred-EMS, a subcellular localization predictor. Protein subcellular localization into endomembrane and secretory pathway classes is predicted by SCLpred-EMS.	[54]
**Microkits 4.0**	http://microkit.biocuckoo.org/	Proteins that temporally and spatially localize to various subcellular locations during cell division/mitosis, such as the centrosome, kinetochore, telomere, and mitotic spindle, are included in MiCroKiTS 4.0.	[55]
**Cello2go**	http://cello.life.nctu.edu.tw/cello2go/	An online platform to examine different features of target proteins and their subcellular positioning	[56]
**Targetp 2.00**	http://www.cbs.dtu.dk/services/TargetP-2.0/	N-terminal sorting signals that direct proteins to the secretory pathway, mitochondria, chloroplasts, or other plastids have been identified using a modern, novel technique.	[57]
**Deeploc**	http://www.cbs.dtu.dk/services/DeepLoc	A prediction algorithm that primarily relies on sequence information to predict the subcellular location of proteins using deep neural networks.	[58]
**MULocDeep**	http://mu-loc.org	Multiple protein localizations at the cellular and sub-organelle levels can be predicted using MULocDeep, a general deep learning-based localization prediction framework.	[59]
**DBSubLoc**	http://www.bioinfo.tsinghua.edu.cn/dbsubloc.html	The primary protein databases, model organism genome projects, and literary texts were used to create a protein sub-cellular localization annotation database. After that, an analysis was conducted to identify the proteins' subcellular localization characteristics and categorize them.	[60]
**Wolf psort**	https://wolfpsort.hgc.jp/	Based on sorting signals, amino acid composition, and functional motifs like DNA-binding motifs, WoLF PSORT transforms protein amino acid sequences into numerical localization features. Following conversion, prediction is done using a straightforward k-nearest neighbour classifier.	[61]
**Busca**	http://busca.biocomp.unibo.it/	a novel web server that combines multiple computational tools to forecast proteins' subcellular locations. BUSCA combines techniques for differentiating between the subcellular localization of globular and membrane proteins with tools for identifying GPI anchors, transmembrane domains, and signaling and transit peptides.	[62]

#### 3.3.1 Critical analysis of subcellular localization prediction methods

Among the servers that can predict subcellular localization, the main limitation is that they cannot predict subcellular localization for proteins from all organisms, including Gram-negative bacteria, Gram-positive bacteria, viruses, Archaea, and others. Additionally, they are unable to process a large number of proteins simultaneously. Among the servers we reviewed, **PSORTb** for bacteria and Archaea is particularly useful and comprehensive, while **Cell-PLoc** can be helpful for virus protein localization predictions.

#### 3.3.2 Future research directions

We suggest developing a server with the following capabilities.

• Support for all types of organisms.
• Ability to process a large number of proteins simultaneously.
• Generation of results in a user-friendly output format, such as an Excel file.

### 3.4 Post-translational modification (PTM) and signal peptide

After translation, proteins may undergo many changes under the influence of various chemical reactions. These changes may be necessary to increase the life of the protein, for its normal activity, or the localization of the protein in the cell. All these changes occurred in the process that is called Post-translational modification (PTM).

During that process, properties of a protein change through activities like cleaving the protein, adding a modification group to one or some amino acids, and so on [63]. PTM plays an important role in many biological processes by significantly impacting protein structure and dynamics [64]. It happens in single or multiple amino acids and leads to changes in the chemical Features of the modified sites [65].

**Fig. 4:**
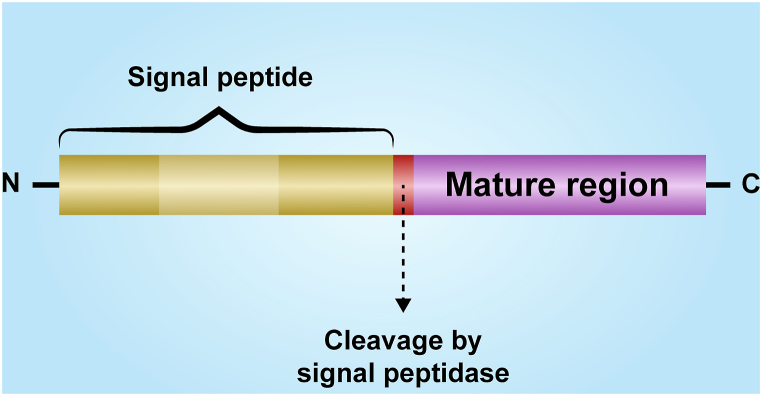
Signal peptide.

As depicted in Fig. 4, Signal peptides (SPs) are short peptides, carrying information for protein secretion or integration into cell membranes [66]. The role of signaling peptides in a specific protein is that, with this targeting sequence, the location in different organelles and the specific secretory pathway for that protein are determined [64]. They are ubiquitous in all prokaryotes and eukaryotes. The structure of SPs consists of three parts: the hydrophilic N- and C-terminal sections on each side of the center hydrophobic h region [67].

SPs incorporated into the ER membrane after protein translation is finished are frequently removed from the final protein by specialized signaling peptidase (SPase) following post-translational modification (PTM) [68]. So, because in the process of PTM, the signal peptides are removed, we should find the signal peptides of our proteins that we found until this part and remove them. For this purpose, we have lots of servers, and the list of the important ones is in Table 3.

**Table 3:** list of signal peptide prediction servers.

Server	URL	Description	Ref
SignalP 5.0	http://www.cbs.dtu.dk/services/SignalP	A deep neural network-based method called Signalp.5 helps to identify three different types of sps in prokaryotes and increases the predictability of sps across all domains of life.	[69]
Phobius	http://phobius.cgb.ki.se/ http://phobius.binf.ku.dk/	A Markov model web server called Phobius combines predictions of signal peptides with transmembrane topology. The approach allows constrained and homology-enriched predictions and selects transmembrane segments and signal peptides in the best possible way.	[70]
PrediSi	http://www.predisi.de/	A novel tool for correctly predicting signal peptide sequences and the locations of their cleavage in bacterial and eukaryotic amino acid sequences is particularly helpful for real-time, large-scale dataset analysis.	[71]
Signal-3L 3.0	http://www.csbio.sjtu.edu.cn/bioinf/Signal-3L/.	A 3-layer hybrid method that integrates deep learning algorithms and window-based scoring is used in Signal-3L 3.0, an improved approach for signal peptide recognition and cleavage-site prediction.	[72]
deepsig	https://deepsig.biocomp.unibo.it/	An improved approach based on deep learning techniques for signal peptide detection and cleavage site prediction.	[73]
EMBOSS Sigleave	http://bio.biomedicine.gu.se/cgi-bin/emboss/sigcleave	sigcleave uses the von Heijne method to predict the location of cleavage between a mature exported protein and a signal sequence.	
Pred-signal	http://bioinformatics.biol.uoa.gr/PRED-SIGNAL/	a hidden Markov model process that predicts the existence of SPs and their cleavage sites and separates them from cytosolic and transmembrane proteins is developed by an archaeal signal peptide prediction server.	[74]
SOSUI signal	https://www.tuat.ac.jp/mitaku/sosui/	The development of SOSUI allowed for the prediction of transmembrane helices as well as the differentiation between soluble and membrane proteins.	[75]
LipoP	http://www.cbs.dtu.dk/services/LipoP/	The LipoP method has been developed to predict lipoprotein signal peptides in Gram-negative Eubacteria.	[76]
Speplip	http://gpcr.biocomp.unibo.it/predictors/	a neural network-based technique, developed and tested on a set of experimentally derived signal peptides from eukaryotes and prokaryotes, detects the presence of sorting signals and predicts their cleavage sites.	[77]
Pred-tat	http://www.compgen.org/tools/PRED-TAT/	The technique, which is based on hidden Markov models, is qualified for differentiating between Sec and Tat signal peptides and predicting their cleavage sites. It also maintains a modular architecture that is appropriate for both types of peptides.	[78]

#### 3.4.1 Critical analysis of signal peptide prediction methods

Among the servers for signal peptide prediction, the main issue is that many of them cannot work with all types of organisms and do not produce graphical outputs. However, **SignalP Version 5** is the most effective for working with eukaryotes, Gram-negative, and Gram-positive bacteria, while **SignalP Version 6** is particularly useful for virus prediction. These servers can handle large numbers of sequences simultaneously, allow users to input multiple sequences at once, enable organism selection, and provide outputs in various formats, including graphical representations.

### 3.5 Transmembrane helix prediction

Transmembrane proteins are essential membrane proteins that extend the whole cell membrane. They play a key role in numerous essential cell processes, such as membrane trafficking, protein targeting, intracellular transport, ion and molecule transport across impermeable membranes, and receptor and signaling transduction pathway [79].

**Fig. 5:**
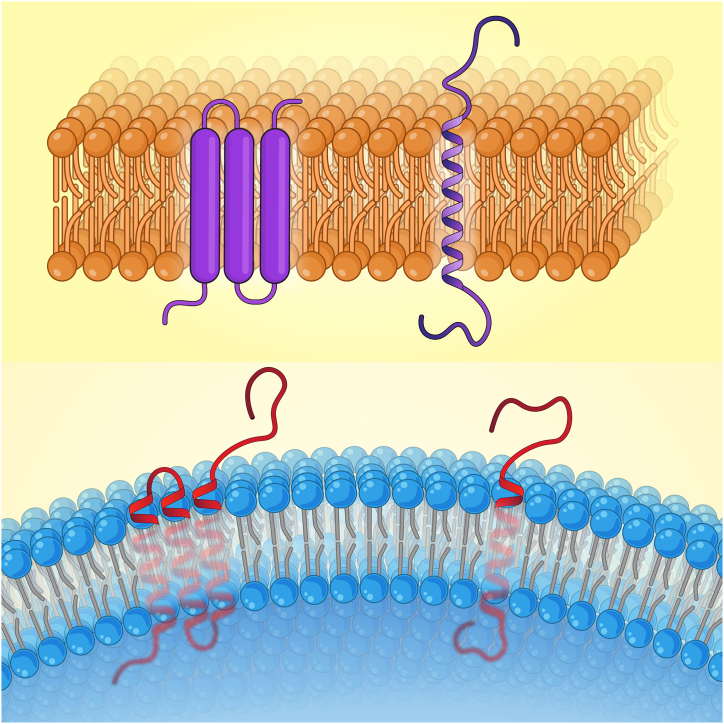
transmembrane helix.

To increase the accuracy of the research, the desired antigens should be examined in terms of their location inside or outside the membrane. As depicted in Fig. 5, in comparison to the sequences inside the membrane, sequences outside the membrane are considered better antigens.

Thus, it is preferable to remove the intramembrane section of the antigen and use only the section that is outside the membrane. The servers described in Table 4 are available for predicting transmembrane helix. Among the available tools, we found **TMHMM** to be one of the best servers. It is highly reputable, with good time complexity and speed. It can generate results in both text and graphical formats. Additionally, it supports up to 10,000 sequences and 4,000,000 amino acids per submission.

**Table 4:** list of transmembrane helices prediction servers.

Server	URL	Description	Ref
TMHMM	http://www.cbs.dtu.dk/services/TMHMM/	a new hidden Markov model-based membrane protein topology prediction technique called TMHMM.	[80]
HMMtop	http://www.enzim.hu/hmmtop	The HMMTOP server is used to predict the topology of transmembrane proteins and to localize helical transmembrane segments.	[81]
TmPred	http://www.ch.embnet.org/software/TMPRED_form.html	The orientation of membrane-spanning sections is predicted by the Transmembrane Predictor web service (TMPred).	[82]
DAS	https://tmdas.bioinfo.se/	The dense alignment surface (DAS) technique is designed to analyze prokaryotic membrane proteins' transmembrane alpha-helices. Forecast.	[83]
TM Finder	http://www.bioinformatics-canada.org/TM/	a transmembrane protein segment prediction program that combines scales of hydrophobicity and nonpolar phase helicity.	[84]

### 3.6 Antigenicity

A protein's capacity to trigger an immune reaction in the human body upon exposure is known as its antigenicity. The terms "allergenicity" and "immunogenicity" refer to two different categories of immune characteristics [85].

#### 3.6.1 Immunogenicity

The ability of an antigen to trigger both innate and adaptive immune responses after coming into contact with the human body is known as immunogenicity. The proteins considered as candidates for vaccine development must possess immunogenic characteristics. Immunogenicity can be calculated on different servers; on these servers, the immunogenicity of proteins is scored, and based on a certain threshold, these proteins are classified as antigens or non-antigens. The servers that perform this prediction are listed in Table 5. Among the available tools, **VaxiJen: Prediction of Protective Antigens and Subunit Vaccines** is one of the best. It allows users to input all sequences in a single run, and you can select the target organism. The server is fast in delivering results. However, one limitation is that the output file is not well-organized, and sometimes you need to separate each line manually using programming languages. Despite this, it remains one of the best tools available to date.

**Table 5:** list of some immunogenicity prediction servers.

Server	URL	Description	Ref
VaxiJen	http://www.jenner.ac.uk/VaxiJen	a novel method for alignment-free antigen prediction that creates uniform vectors of significant amino acid characteristics (ACC) from protein sequences.	[86]
ANTIGENpro	http://scratch.proteomics.ics.uci.edu/	A web server that combines high-throughput technologies with information gleaned from the literature and preexisting databases to create a sizable and non-redundant library of antigens with established immunogenicity.	[87]
NetMHCpan	http://www.cbs.dtu.dk/services/NetMHCpan-4.1/	An effective method for predicting the specificity of a T-cell immune response is to predict peptide binding to MHC. This served as inspiration for the development of two web servers, NetMHCpan-4.1 and NetMHCIIpan-4.0, which predict the binding of peptides to MHC-I and MHC-II.	[88]
Jenner-predict	http://117.211.115.67/vaccine/home.html	A web server has been created to predict PVCs based on bacterial pathogen proteomes. It identifies non-cytosolic proteins with recognized functional domains from protein classes including virulence, adhesin, invasin, porin, flagellin, colonization, toxin, transferring, choline, penicillin, and solute binding as PVCs.	[89]
Pcleavage	http://www.imtech.res.in/raghava/pcleavage/ http://bioinformatics.uams.edu/mirror/pcleavage/	The main cellular mechanism responsible for the degradation of intracellular proteins is the proteasome, which also generates peptides that can attach to MHC class I molecules. Since MHC class I ligands with proteasome cleavage sites at their C-termini are known to be more likely to be T-cell epitopes, predicting the sites of cleavage is essential for the development of subunit vaccines.	[90]

#### 3.6.2 Allergenicity

The term "allergenicity" describes an antigen's capacity to trigger an abnormal immune response, an overreaction that reduces physiological function or damages tissue. Since the allergenicity of a protein carries risks, including death, it is necessary to find the allergenic proteins and remove them from the desired sequences. The servers that perform this prediction are listed in Table 6. Among the available tools for predicting allergenic potential, many use simple classifier algorithms like KNN, AAC-based RF, Hybrid (RF + BLAST + MERCI), and others, often with limited datasets. We suggest developing additional servers that can leverage more advanced methods, such as Artificial Neural Networks (ANNs), to improve accuracy and prediction capabilities. Another limitation is that many servers cannot process large numbers of proteins simultaneously, requiring researchers to submit them one by one. However, among these tools, **AllerTOP** and **AllergenFP** stand out as particularly useful due to their speed and user-friendly interfaces.

**Table 6:** list of some allergenicity prediction servers.

Server	URL	Description	Ref
AlgPred 2.0	https://webs.iiitd.edu.in/raghava/algpred2/	AlgPred 2.0 is a web server that facilitates allergen prediction, IgE epitope mapping, motif search, and BLAST search.	[91]
SDAP	http://fermi.utmb.edu/SDAP/	Using an original algorithm based on conserved properties of amino acid side chains, a web server that offers fast, cross-referenced access to the sequences, structures, and IgE epitopes of allergenic proteins finds regions of known allergens that are similar to user-supplied peptides or selected from the SDAP database of IgE epitopes.	[92]
AllergenPro	http://nabic.rda.go.kr/allergen/	A complete web-based platform offering details on allergens in food, microbes, animals, and plants.	[93]
Allergen Online	https://www.allergenonline.org/	a platform for peer-reviewed bioinformatics to assess the risks associated with novel foods and genetically modified organisms (GMOs) that contain new dietary proteins.	[94]
AllergenFP	http://ddg-pharmfac.net/Allergen FP	A four-step algorithm that uses a novel alignment-free descriptor-based fingerprint approach to distinguish between allergens and non-allergens is used.	[95]
Allermatch	https://www.allermatch.org/	is an online tool that allows users to quickly and accurately predict whether novel proteins, like genetically modified organisms (GMOs), will cause allergies in proteins or peptides.	[96]
AllerTOP v2	http://www.ddg-pharmfac.net/AllerTOP	various machine learning techniques for classification, including logistic regression (LR), decision trees (DT), naive bayes (NB), random forests (RF), multilayer perceptrons (MLP), and k nearest neighbours (kNN), as well as amino acid E-descriptors and auto- and cross-covariance transformation	[97]
EVALLER	http://bioinformatics.bmc.uu.se/evaller.html	The "Detection based on Filtered Length-adjusted Allergen Peptides" (DFLAP) algorithm-based web server facilitates fast analysis of query amino acid sequences. With excellent sensitivity and specificity, it offers in silico detection of potential protein allergenicity in FASTA format.	[98]
SPADE	https://spade.uni-graz.at/	A web server that differentiates between potential IgE-binding sites and non-cross-reactive surface regions using the structural homology of cross-reactive allergens and immunological cross-reactivity data	[99]
PREAL	http://gmobl.sjtu.edu.cn/PREAL/index.php	The maximum relevance minimum redundancy (mRMR) method and incremental feature selection (IFS) are the foundations of this web application. These techniques were used to integrate different protein properties, such as biochemical and physicochemical properties and sequential and subcellular locations, to obtain essential features for allergenicity.	[100]

### 3.7 Toxicity

Vaccines improve immunity against diseases, but first of all, we should check the efficacy, quality, and safety of them [101]. Preclinical toxicological investigations are necessary before beginning and concurrently with clinical research to ensure the efficacy of novel vaccinations. In this kind of research, where we have lots of antigens for constructing a vaccine, each should be checked for toxicity, and those likely to be toxic should be removed from the project.

We should choose non-toxic antigens for our research. The servers described in Table 7 are available for toxicity prediction. Among the available tools, **ToxinPred** is highly reputable with a complete database and uses an SVM classifier. Its speed in predicting toxicity is impressive. However, it cannot process peptides shorter than 10 amino acids in length. In such cases, you should use the "Design Peptide" tab to work with peptides shorter than 10 residues.

**Table 7:** list of toxicity prediction servers.

Server	URL	Description	Ref
Toxin-pred	http://crdd.osdd.net/raghava/toxinpred/	A web server called ToxinPred was created to predict peptide toxicity or non-toxicity, the minimum amount of peptide mutations, and the toxic regions of proteins. It has been noted that toxic peptides contain high concentrations of specific residues, including Cys, His, Asn, and Pro.	[102]
NNTox	http://kiharalab.org/nntox_dataset/	GO terms and protein toxicity are analyzed in this work to develop predictor models of protein toxicity and to extend general function prediction methods for predicting the toxicity of proteins, such as a neural network model and multi-label model.	[103]
ClanTox	http://www.clantox.cs.huji.ac.il/	A tool for predicting the discovery of toxin-like cell modulators—many of which are potential therapeutic agents—is called ClanTox (Classifier of Animal Toxins). It is predicated on taking the primary protein sequence and extracting sequence-driven features, then using a classification system that has been trained on known animal toxins.	[104]
SpiderP	http://www.arachnoserver.org/spiderP.html	A common source of novel compounds with potential applications in medicine and agrochemistry are spider neurotoxins, which are also employed as pharmacological tools. Better than or on par with existing methods for predicting propeptide sequences in spider toxins is SpiderP, a support vector machine (SVM) framework that integrates local and global sequence information.	[105]

### 3.8 Blast and homology

There is a fundamental fact that the immune system does not produce immunity against host proteins. Therefore, in the process of designing a multi-epitope vaccine, if researchers choose an antigen similar to host proteins, it implies that the immune system cannot generate immunity against it, or the immunity produced may not be optimal. Hence, researchers in this field must select antigens that are dissimilar to any proteins of host cells. The server capable of verifying this aspect is Blast, whose address is https://blast.ncbi.nlm.nih.gov/Blast.cgi. On this website, you can identify any proteins similar to your antigens. If there is any resemblance between host proteins and your antigens, it signifies that the chosen antigen may not be suitable for the research. It's important to note that the cutoff values must be: >30 % identity with host proteins, E-value <0.005, and query coverage ≥70 %, indicating meaningful similarity.

### 3.9 B- and T-cell Epitope prediction

As clarified in previous sections, after downloading the genome of the target for which we aim to design a multi-epitope vaccine, we need to identify all available Open Reading Frames (ORFs) in the genome. Subsequently, we apply various filters to these ORFs to pinpoint the best antigens. Upon reaching this stage, you will encounter antigens that have successfully passed all filters and steps.

It is recommended to choose an antigen with a high antigenicity score. Once the best antigen is selected, researchers can present it as one of the research outputs, as they identify this antigen as a potential candidate in vaccine research projects against the target disease. Other researchers can also use this antigen in their projects.

Following this, we can use the best antigen to identify available epitopes and proceed with the design of a multi-epitope vaccine against the target disease. There are several different servers for predicting the epitopes of B and T cells epitopes, which are introduced in Table 8.

**Table 8:** epitope prediction servers.

Servers	URL	Description	Ref
**IEDB**	http://www.iedb.org/	A comprehensive database of B-cell and T-cell epitopes from nonhuman primates, humans, and other animals that have been determined through experimentation.	[106]
**People**	http://leps.cs.ntou.edu.tw/	A novel B-cell LE prediction system	[107]
**BepiPred**	http://www.cbs.dtu.dk/services/BepiPred/	Linear B cell epitope prediction	[108]
**ABCpred**	http://www.imtech.res.in/raghava/abcpred/	Predictor of linear B cell epitope using artificial neural network.	[109]
**LBtope**	http://www.imtech.res.in/raghava/lbtope/	ca web server for B-cell epitope design and prediction.	[110]
**BCEPred**	http://www.imtech.res.in/raghava/bcepred/	Linear B cell epitopes prediction by physicochemical properties	[111]
**BEPITOPE**	jlpellequer@cea.fr	A technique that uses sequences to predict continuous B cell epitopes.	[112]
**COBEpro**	http://scratch.proteomics.ics.uci.edu	An innovative two-phase method for predicting continuous B-cell epitopes.	[113]
**EPMLR**	http://www.bioinfo.tsinghua.edu.cn/epitope/EPMLR/	sequence-based linear B-cell epitope prediction method.	[114]
**DRREP**	https://github.com/gsher1/DRREP	A newly developed, synthesized, and analytically trained core deep neural network Deep Ridge Regressed Epitope Predictor (DRREP) provides for continuous epitope prediction use.	[115]
**FBCPred**	http://ailab.cs.iastate.edu/bcpreds/	a unique technique that uses the subsequence kernel to predict flexible-length linear B-cell epitopes.	[116]
**BCPREDS**	http://ailab.cs.iastate.edu/bcpreds/	a unique technique that uses the subsequence kernel to predict linear B-cell epitopes.	[117]
**AAPPred**	https://www.bioinf.ru/aappred/	Based on the discovery that certain amino acid pairs (AAPs) are preferred by B-cell epitopes, a brand-new scale known as the amino acid pair (AAP) antigenicity scale is presented.	[118]
**CBTOPE**	http://www.imtech.res.in/raghava/cbtope/	A method for conformational B cell epitope prediction using antigen primary sequence.	[119]
**CEP**	http://bioinfo.ernet.in/cep.htm	An online interface for the method for predicting conformational epitopes.	[120]
**DiscoTope**	http://tools.iedb.org/discotope/	a technique for forecasting discontinuous epitopes from protein 3D structures in PDB format.	[121]
**PEPITO**	http://pepito.proteomics.ics.uci.edu/	Structure-based approach (geometric structure and physicochemical attributes)	[122]
**PEPITOPE**	http://pepitope.tau.ac.il/	an online application designed to forecast discontinuous epitopes.	[123]
**EpiDOCK**	http://epidock.ddg-pharmfac.net	first structure-based server for predicting MHC class II binding.	[124]
**IEDB-MHCI**	http://tools.immuneepitope.org/mhci/	a collection of online resources for immunological epitope analysis and prediction.	[125]
**IEDB-MHCII**	http://tools.immuneepitope.org/mhcii/	a collection of online resources for immunological epitope prediction and analysis.	[125]
**NetMHC**	http://www.cbs.dtu.dk/services/NetMHC/	T-cell class I epitope prediction using an improved neural network technique.	[126]
**NetMHCII**	http://www.cbs.dtu.dk/services/NetMHCII/	MHC–II–peptide binding affinity prediction methods.	[127]
**MHCPred**	http://www.ddg-pharmfac.net/mhcpred/MHCPred/	IC50 values for TAP/peptide or MHC/peptide.	[128]
**IEDB binding**	http://www.immuneepitope.org/analyze/html/mhc_processing.html	epitopes of CD8 + T cells TAP binding and cleavage sites.	[129,130]
**Rankpep**	http://imed.med.ucm.es/Tools/rankpep.html	Prediction of MHC I and II binding peptides.	[131,132]
**EpiTOP**	http://www.pharmfac.net/EpiTOP	MHC class II binding using a proteo-chemometric tool.	[133]
**CTLpred**	https://webs.iiitd.edu.in/raghava/ctlpred/	ANN and SVM-based CTL epitope prediction.	[134]
**TEPITOPE**	http://datamining-iip.fudan.edu.cn/service/TEPITOPEpan/ TEPITOPEpan.html	Prediction of promiscuous MHC class 2 epitopes	[135]
**Pcleavage**	http://www.imtech.res.in/raghava/pcleavage	SVM-based approach for predicting proteasome cleavage.	[90]
**IL4pred**	http://webs.iiitd.edu.in/raghava/il4pred/index.php	SVM-based method for predicting IL4 inducing peptides.	[136]
**NetMHCpan**	http://www.cbs.dtu.dk/services/NetMHCpan/	service uses artificial neural networks (ANNs) to predict how peptides will bind to any MHC molecule with a specified sequence.	[137]
**NetMHCIIpan**	http://www.cbs.dtu.dk/services/NetMHCIIpan/	MHC–II–peptide binding ffinity prediction methods.	[127]
**NetChop**	http://www.cbs.dtu.dk/services/NetChop	Human proteasomes' cleavage sites.	[138]
**PREDEP**	http://margalit.huji.ac.il	MHC I epitope prediction	
**ProPred**	http://www.imtech.res.in/raghava/propred/	A peptide prediction server for MHC II binding.	[139]
**ProPred-1**	http://www.imtech.res.in/raghava/propred1	A peptide prediction server for MHC I binding.	[140]
**TAP Pred**	http://www.imtech.res.in/raghava/tappred	Predict the peptide's affinity for binding to the TAP transporter.	[141]

#### 3.9.1 Critical analysis of Epitope prediction methods

Among the available tools for epitope prediction, several servers have their strengths and weaknesses. For linear B-cell epitopes, servers like **BepiPred**, **ABCpred**, and **BCEPred** are commonly used. **BepiPred** is known for its ability to predict continuous B-cell epitopes based on sequence information, but it doesn't work well with all types of epitopes, especially conformational ones. **ABCpred** uses an artificial neural network for linear B-cell epitope prediction, offering high accuracy, but it may not handle larger datasets effectively. **BCEPred**, based on physicochemical properties, is useful for predicting linear epitopes, but its predictions are limited to certain amino acid sequences and may not always be as precise for complex structures.

In the case of conformational epitopes, **DiscoTope** and **PEPITO** are highly regarded. **DiscoTope** predicts discontinuous epitopes from 3D structures in PDB format, which is useful for studying the spatial arrangement of epitopes but requires access to high-quality structural data. **PEPITO**, which uses both geometric and physicochemical properties, is effective for structure-based prediction but can be limited by the availability of suitable 3D structures.

For T-cell epitope prediction, tools like **NetMHC**, **MHCPred**, and **IEDB-MHC** are widely used. **NetMHC** offers a reliable prediction of MHC class I binding affinity using a neural network, but it may struggle with some MHC alleles or non-human species. **MHCPred** provides IC50 values for TAP/peptide or MHC/peptide binding, but its performance can be inconsistent with certain peptide sequences. **IEDB-MHC** provides a comprehensive suite of resources for T-cell epitope prediction, covering a wide range of MHC alleles and species, but may have slower performance with large datasets.

Although these tools each have their advantages, such as **IEDB** offering the broadest coverage for multiple organisms, the main drawback across many servers is their limited ability to process large datasets simultaneously, requiring users to submit sequences one by one. Additionally, the output files from some servers may not always be well-organized, requiring additional processing or coding to clean up the results.

Considering all factors, we recommend **IEDB** as the most comprehensive and versatile tool for epitope prediction. Despite its slower processing speed with larger datasets, it allows for a wide range of selections, including various target organisms and epitope lengths. It is well-established, offers robust support for both B-cell and T-cell epitopes, and is frequently used by researchers globally.

### 3.10 Linker and adjuvant selection

After predicting all epitopes, it is recommended to assess their antigenicity scores using servers such as Vaxijen. In this scenario, you can organize epitopes based on their antigenicity scores, ultimately selecting those with the highest scores as your final candidates. It is important to note that considering toxicity and allergenicity is crucial.

Therefore, even if an epitope has a high antigenicity score, it is suitable for use in a vaccine candidate only if it is non-toxic and non-allergenic. Once the best epitopes have been chosen, they can be assembled into a multi-epitope vaccine.

Constructing a vaccine necessitates the incorporation of linkers and adjuvants. Toll-like receptors (TLRs), immune receptors, play a pivotal role in activating the body's defenses against bacterial and viral infections [142].

Adjuvants, whether biological or chemical structures, elicit innate and adaptive immune responses when interacting with TLRs, making them valuable in vaccine design [143]. Commonly used adjuvants in multi-epitope vaccine design include.

• Beta-actin [144].
• Beta-defensin 3 [145].
• Human Beta-defensin 2 [146].
• Human Beta-defensin 3 [147].
• Cholera toxin subunit B [148].
• CpG-containing, Hp91 [149].
• Hemagglutinin [150].
• oligodeoxynucleotides (IMODNs) [151].
• class B [152].
• heparin-binding hemagglutinin adhesin (HBHA) [153].
• 50S ribosomal protein L7/L12 [154].
• Cholera Toxin B, CTxB [148,155].
• Escherichia coli heat-labile enterotoxin B subunit (LTB) [156].
• diphtheria toxin, DT386 [157].
• Toll-like receptors (TLRs) [158].
• and ect.

Linkers are brief amino acid chains that can separate individual sequences essential for the structural stability, cross-domain interactions, and operation of vaccines [167]. To decrease junctional immunogenicity and enable effective separation and presentation to B and T cells, they were inserted in between epitopes [168].

The selection of a linker is dependent on various factors such as length, amino acid sequence, hydrophobicity, secondary structure, susceptibility to proteases, and potential interactions with other components of the immunogenic construct.

Although they serve different functions, the linkers KK, AAY, GPGPG, and EAAAK are often used. It is recommended that CTL epitopes be linked using the "AAY" linker. HTL and BC epitopes should be connected using the "GPGPG" and "KK" linkers. It is recommended that the adjuvant be linked to the vaccine construct using a rigid linker sequence such as 'EAAAK' to enhance protein stability [159].

### 3.11 Vaccine validation

When you reach this section, a candidate for an epitope-based vaccine has been designed. Now, it is necessary to assess the validity and effectiveness of this vaccine. In this section, we will introduce important analyses that the vaccine candidate must undergo and be evaluated for.

#### 3.11.1 Secondary and tertiary structure

Proteins are linear chains of amino acids that perform various functions. However, during their lifecycle, proteins fold into appropriate spatial conformations for the biological functions they provide. The primary protein structure is determined by the arrangement of amino acids and the locations of disulfide bonds [160]. In contrast, the secondary protein structure refers to the regular, local structure of the protein backbone [161].

The spatial arrangement of the elements of a protein's secondary structure forms its tertiary structure, or fold. The tertiary structure is held together by non-covalent interactions such as disulfide bonds, hydrophobic packing, ionic contacts, hydrogen bonding, van der Waals forces, and coordination of metal ions [162].

Because alpha-helix has little antigenic properties, a significant quantity of it in a recombinant protein can complicate synthesis [163].

A secondary structure study should be conducted to guarantee that the vaccination candidate does not include an excessive amount of alpha helices. An important note is that in future steps, we should know the predicted 3rd structure of the vaccine to evaluate and use it for structural analyses like docking and molecular dynamics.

Therefore, it is crucial to predict both the 2nd and 3rd structures of the vaccine candidate. Various servers for identifying and analyzing proteins' secondary and tertiary structures are listed in Table 9.

**Table 9:** Protein secondary & tertiary structure prediction servers.

Server	URL	Ref
**Secondary structure prediction servers**		
PSIPRED	http://bioinf.cs.ucl.ac.uk/psipred/	[164]
JPred4	(http://www.compbio.dundee.ac.uk/jpred4)	[165]
GOR V	http://gor.bb.iastate.edu/	[166]
Porter	http://distill.ucd.ie/porter/	[167]
trRosetta	https://yanglab.nankai.edu.cn/trRosetta/https://yanglab.nankai.edu.cn/trRosetta/download/	[168]
BeStSel	https://bestsel.elte.hu	[169]
RaptorX	http://raptorx2.uchicago.edu/StructurePropertyPred/predict/	[170]
PredictProtein	https://predictprotein.org)	[171]
**Tertiary structure prediction servers**		
Robetta	http://robetta.bakerlab.org/	[172]
YASARA	http://www.yasara.org/	[173]
FALCON2	http://protein.ict.ac.cn/FALCON2	[174]
AWSEM	https://awsem.rice.edu	[175]
ProTSAV	http://www.scfbio-iitd.res.in/software/proteomics/protsav.jsp	[176]
MULTICOM2	https://github.com/multicom-toolbox/multicom/tree/multicom_v2.0	[177]
HHpred	https://toolkit.tuebingen.mpg.de/tools/hhpred	[178]
SWISS-MODEL	http://swissmodel.expasy.org/	[179]

#### 3.11.2 Ramachandran plot

In the previous section, we predicted the third structure of our vaccine, and now we can conduct a structural analysis. One essential aspect of this analysis is the Ramachandran plot, which evaluates the Phi and Psi angles. Scientists commonly employ this plot to scrutinize the conformations of amino acids in proteins [180]. The Ramachandran diagram illustrates the permissible states for each angle in protein structures. Refer to Table 10 for a list of various servers available for generating Ramachandran plots.

**Table 10:** Ramachandran plot servers.

Server	URL	Ref
**P**rocheck	https://www.ebi.ac.uk/thornton-srv/software/PROCHEC	[181]
MolProbity	http://molprobity.biochem.duke.edu/	[182]
PDBsum	http://www.ebi.ac.uk/pdbsum	[183]
Rama	http://inctipp.bioagro.ufv.br:8080/Rama	[184]
VADAR	http://redpoll.pharmacy.ualberta.ca/vadar	[185]

#### 3.11.3 Other structural analysis

Beyond the Ramachandran analysis, there are numerous servers available for evaluating the third structure of your vaccine. One such server is the "UCLA-DOE LAB — SAVES v6.0," accessible at https://saves.mbi.ucla.edu/. Through this server, you can assess the vaccine's structure across various dimensions, including:

**ERRAT (Statistics of Non-Bonded Interactions):** The statistics of non-bonded interactions between various atom kinds are analyzed. It plots the value of the error function against the location of a sliding window with nine residues. To calculate this, statistics from highly refined structures are compared [186].

**VERIFY 3D (Compatibility with Amino Acid Sequence):** finds the correspondence between a 3D atomic model and its corresponding amino acid sequence (1D). Based on the environment and position, it determines the structural class (alpha, beta, loop, polar, nonpolar, etc.) and compares the results with known structures [187,188].

**PROCHECK (Stereochemical Quality Check**: By examining both the overall structural geometry and the geometry of individual residues, it evaluates the stereochemical quality of a protein structure [189,190].

**WHATCHECK (Extensive Stereo-checmical Parameter Checking):** Using a subset of the WHAT IF program's protein verification abilities, this tool analyses the various stereochemical features of the residues in the model [191].

#### 3.11.4 Physical-chemical properties

In this phase, it is advisable to evaluate the physical-chemical properties of the constructed vaccines. The Protparam server, available at https://web.expasy.org/protparam/, is among the best tools for this purpose. In this section, we will highlight some crucial parameters that researchers should examine:

**Molecular Weight:** Usually, larger molecules serve as better antigens compared to smaller ones. Although an exact molecular weight threshold for immunogenicity is challenging to determine, substances must possess a minimum molecular weight. The required minimum is 1000 Da. Substances within the molecular weight range of 1000–6000 Da exhibit an intermediate state. Those exceeding 6000 Da are generally immunogenic.

**Theoretical pI (Isoelectric Point):** The theoretical pI of a protein is the pH at which the net charge becomes zero. Below the pI, proteins carry a positive charge, while above it, they are negatively charged. The protein pI can range from very acidic values, approximately 4.0, to very alkaline values, around 12.0.

**Extinction Coefficients:** Extinction coefficients indicate how much light a protein absorbs at a specific wavelength. This value is valuable for UV spectral analysis studies on the vaccine's structure.

**Instability Index:** The instability index is a standard used to assess protein stability in tube tests. Scores below 40 suggest probable stability, while scores exceeding 40 indicate potential instability.

**Aliphatic Index:** The aliphatic index contributes to the thermal stability of proteins. Higher aliphatic indices correlate with increased thermal stability.

**GRAVY (Grand Average of Hydropathy):** GRAVY is a metric used to indicate the hydrophobicity of a peptide. Positive GRAVY values denote hydrophobicity, while negative values signify hydrophilicity.

#### 3.11.5 Molecular docking and molecular dynamic simulation

In this section, scientists should analyze docking and molecular dynamic simulations. Molecular docking studies involve examining how two biological compounds fit together or interact [192].

In the final stage of vaccine validation, molecular docking should be performed between the vaccine and MHC molecules to determine their connection, fit, and interaction. The PDB ID for MHC1 is 5YXU, and for MHCII, it is 4MD4. These structures are considered optimal candidates, and scientists can dock their vaccine candidates with these structures.

For this purpose, different complex orientations should be investigated to determine which orientation has the highest stability [193]. Docking results are presented as a docking score, the number of amino acids involved in binding, and other criteria. Various servers and tools for molecular docking are listed in Table 11.

**Table 11:** molecular docking SERVERS.

Name	Url	Description	Ref
**Docking software**			
AutoDock	https://vina.scripps.edu/	Automated docking of ligand to macromolecule using Empirical Free Energy Scoring Function and Lamarckian Genetic Algorithm	[194]
Glide	https://newsite.schrodinger.com/platform/products/glide/	Using a high-throughput virtual screening method, ligands are ranked and protein-ligand binding modes are predicted using a ligand docking program.	[195]
Molegro	https://molexus.io/molegro-virtual-docker/	The protein-ligand docking simulation program	[196]
Auto dock vina	https://vina.scripps.edu/	an open-source molecular docking software.	[197]
FlexAID	https://github.com/NRGlab/FlexAID	Soft scoring function based on surface complementarity and target side-chain flexibility.	[198]
LeDock	http://www.lephar.com/download.htm	Software for flexible docking of small molecules into proteins quickly and accurately.	
rDock	https://rdock.github.io/	A molecular docking programme that is available for free that is used to align small molecules with proteins and nucleic acids.	[199]
UCSF DOCK	https://dock.compbio.ucsf.edu/	predicts small molecule binding modes using geometric methods.	[200]
**Docking web servers**			
cluspro	https://cluspro.org/	protein-protein docking	[201]
HDOCK	http://hdock.phys.hust.edu.cn/	a hybrid strategy-based web server for protein-protein and protein-DNA/RNA docking	[202]
CB-Dock	http://cao.labshare.cn/cb-dock/	a web server for protein-ligand blind docking guided by cavity detection	[203]
SwissDock	http://www.swissdock.ch/	protein ligand docking server	[204]
LZerD	https://lzerd.kiharalab.org/	Improved Protein-Protein Docking Webserver Using de novo Structure Prediction	[205]
SeamDock	https://bioserv.rpbs.univ-paris-diderot.fr/services/SeamDock/	A Cooperative and Engaging Web-Based Docking Tool to Support Small Compound Molecular Docking	[206]
LPIcom	http://crdd.osdd.net/raghava/lpicom	A web server for protein-ligand binding site research, comparison, and prediction	[207]
EDock‐ML	http://ml.umsl.edu/	A web server for using ensemble docking with machine learning to aid drug discovery.	[208]
Webina	https://durrantlab.pitt.edu/webina/	an open-source web application and framework that uses the web browser to run AutoDock Vina completely.	[209]
ZDOCK	http://zdock.umassmed.edu/	interactive docking prediction of symmetric multimers and protein-protein complexes.	[210]
COVID-19 docking Server	http://ncov.schanglab.org.cn	a meta server for docking antibodies, peptides, and small compounds against possible COVID-19 targets.	[211]
pyDockDNA	https://life.bsc.es/pid/pydockweb	webserver for protein-DNA docking and scoring based on energy	[212]
HawkDock	http://cadd.zju.edu.cn/hawkdock/	Website to use MM/GBSA and computational docking to predict and analyze the protein-protein complex	[213]

Once the complex of your vaccine-MHC1 and your vaccine-MHC2 is obtained, molecular dynamic simulation analysis can be performed using several software options listed in Table 12. Molecular dynamics (MD) simulations play a crucial role in vaccine design, providing valuable insights into the interactions and dynamic behavior of biological molecules at an atomic level.

**Table 12:** molecular dynamics simulation SERVERS.

Server	Url	description	Ref
ACEMD	https://www.acellera.com/acemd/	A biomolecular dynamics (MD) engine of production class that supports both CHARMM and AMBER force fields is called ACEMD.	[214]
AMBER heard	https://ambermd.org/	A set of force fields called Assisted Model Building with Energy Refinement (AMBER) is used for biomolecule molecular dynamics simulations.	[215]
Abalone	http://www.biomolecular-modeling.com/Abalone/	programme for molecular dynamics and molecular graphics used in biomolecule simulations; primarily used to model DNA-ligand complexes and protein folding in the AMBER force field.	[216]
GROMACS	http://www.gromacs.org/	A comprehensive modelling software that includes various trajectory analysis tools, quick molecular dynamics, normal mode analysis, and essential dynamics analysis for proteins and membrane systems, among other things.	[217]
Avogadro	https://avogadro.cc/	constructing and editing molecules (peptides, small molecules, crystals), conformational analysis, converting between two and three dimensions, and extending interfaces to new tools	[218]
CHARMM	https://www.charmm.org/	For detailed biomolecular modeling	[219]
MDynaMix	https://www.chemcomp.com/	A general-purpose molecular dynamic software program that uses periodic boundary conditions and force fields resembling CHARMM and AMBER to simulate combinations of molecules.	[220]
NWChem	https://nwchemgit.github.io/	Software for high-performance computational chemistry includes combined QM-MM techniques, molecular dynamics, and quantum mechanics.	[221]
Gaussian software	https://gaussian.com/	software for electronic structure simulations.	[222]
NAMD	https://www.ks.uiuc.edu/Research/namd/	Large-scale biomolecular system high-performance simulation using a parallel molecular dynamics code.	[223]
LAMMPS	https://www.lammps.org/	A common tool for modelling materials at length scales ranging from atomic to mesoscale to continuum using particles	[224]
UCSF Chimera	http://www.cgl.ucsf.edu/chimera/	Antechamber and MMTK, visually appealing viewer, amino acid rotamers and other building, and plugins for Amber tools are in development.	[225]
Orac	http://www.chim.unifi.it/orac	Programme for simulating molecular dynamics to investigate free energy surfaces in biomolecular systems at the atomic level.	[226]
Q-Chem	https://www.q-chem.com/	for advanced electronic structure simulations.	[227]
Autodock4	https://autodock.scripps.edu/	Best for molecular docking simulations	[228]
YASARA	http://www.yasara.com/	Molecular graphics, modeling, simulation	[229]

These simulations are extensively used to study the molecular structures, properties, and interactions of potential vaccine candidates with components of the immune system. In vaccine development, the primary goal is to create antigens capable of eliciting a robust and specific immune response against a particular pathogen.

MD simulations enable researchers to explore conformational changes, flexibility, and stability of antigens, helping identify critical regions, known as epitopes, essential for eliciting an immune response. The process of using MD simulations in vaccine design typically involves the following steps:

**Antigen Selection:** Researchers identify potential antigens from the pathogen of interest, which can be proteins, peptides, or other biomolecules crucial for the pathogen's function and virulence.

**Three-dimensional structure:** Structural Modeling Selected antigens' three-dimensional structures are predicted computationally using homology modelling or obtained experimentally using techniques like X-ray crystallography and NMR spectroscopy.

**MD Simulation Setup:** The antigen structure is placed in a simulated environment, often a water box, to mimic physiological conditions. Force field parameters describing interactions between atoms are assigned to the molecules.

**Simulation Execution:** The MD simulation is carried out using powerful computational resources. The simulation tracks the movements of atoms and molecules over time, allowing antigens to adopt different conformations and explore their dynamic behavior.

**Interaction Studies:** During the simulation, researchers can study how antigens interact with various immune system components, such as antibodies or major histocompatibility complexes (MHCs). This information helps identify potential binding sites and epitopes recognized by the immune system.

**Vaccine Optimization:** Based on insights gained from MD simulations, researchers can modify the antigen's structure or sequence to enhance its immunogenicity. They can introduce mutations or use bioinformatics tools to select regions more likely to trigger a robust immune response.

##### 3.11.5.1 Modeling and simulation analysis

After performing molecular dynamics (MD) simulations, the results should be analyzed using various criteria. The most important ones are summarized below.

a) Structural Stability:
  • Root Mean Square Deviation (RMSD): Evaluates the stability of the structure over time.
  • Root Mean Square Fluctuation (RMSF): Analyzes the flexibility of different regions of the vaccine.
  • Radius of Gyration (Rg): Assesses the compactness or expansion of the structure.
b) Interactions with the Immune System:
  • Docking and MM-PBSA/MM-GBSA: Analyze the interactions between the vaccine and immune system receptors (e.g., MHC-II, TLR). Calculate binding energy.
  • Hydrogen Bond Analysis: Identify the number and stability of hydrogen bonds.
  • Salt Bridge Analysis: Examine salt bridges formed between the vaccine and receptors.
c) Dynamics and Flexibility:
  • Essential Dynamics: Analyze collective motions within the molecule.
  • Secondary Structure Analysis: Examine changes in secondary structures (e.g., alpha-helices, beta-sheets).
d) Energy Analysis:
  • Potential Energy: Track changes in the system's potential energy over time.
  • Interaction Energy: Calculate van der Waals and electrostatic interaction energies.
e) Thermal Stability Assessment:
  • Temperature Stability Analysis: Simulate the vaccine at different temperatures to evaluate its thermal stability.
f) Solvent-Accessible Surface Area (SASA):
  • Measure the exposure of different regions of the vaccine to the solvent. This is crucial for predicting antigenicity.
g) Bonding Network:
  • Analyze the molecular bonding network (e.g., hydrogen bonds, salt bridges) during the simulation.
h) Dynamics of Interaction Pathways:
  • Investigate possible pathways for signal transmission between the vaccine and receptors.
I) MM-PBSA/MM-GBSA Analysis:
  • Objective: Calculate the binding free energy between the vaccine and immune system receptors.
  • Components of Binding Free Energy: Molecular Mechanical Energy Includes van der Waals energy and electrostatic energy
  • Polar Solvation Energy: Calculated using the Poisson-Boltzmann (PB) equation or Generalized Born (GB) method.
  • Nonpolar Solvation Energy: Estimated based on solvent-accessible surface area (SASA).

#### 3.11.6 Simulating immune response to the vaccine

In this stage, researchers should stimulate the immune response to the designed vaccine using computational tools that mimic the real body environment. Various tools are available for this purpose, but based on our review, we can highlight SnapGene as the most suitable software for simulating the cloning process of the codon-optimized vaccine into the pET28(+) vector. This tool is widely recognized for its speed, efficiency, and ease of use, making it an ideal choice for simulating vaccine cloning. SnapGene also plays a crucial role in optimizing the design of the vaccine, offering insights into the cloning process before experimental validation. While other tools are available for simulating immune responses and interactions, SnapGene stands out due to its robust performance and high level of recognition in the scientific community.

## 4 Discussion

The *COVID-19* pandemic has underscored the crucial need for vaccine development in contemporary times. To effectively respond to health emergencies and mitigate the mortality rates of infectious diseases, it is imperative that scientists can rapidly design vaccines against various bacteria, viruses, and other foreign pathogens that can cause illnesses upon entering the body. However, conventional vaccine design processes are characterized by being time-consuming and costly. Nevertheless, the promising news is that bioinformatics and computational techniques can assist in reducing the time and cost associated with these endeavors. Bioinformatics has revolutionized vaccine design, particularly with advancements in genome sequencing and computational modeling.

By leveraging computational tools and high-throughput genomic data, researchers can now rapidly identify and design effective vaccine candidates, overcoming many of the challenges associated with traditional vaccine development. Computational tools, such as molecular modeling and bioinformatics, facilitate the design and optimization of vaccine candidates, predicting their immunogenicity and stability. Machine learning algorithms further assist in analyzing vast datasets to uncover immune system responses, while in silico simulations reduce reliance on costly and time-intensive experimental trials. These advancements significantly shorten development timelines and enhance the adaptability to emerging infectious diseases.

This review discusses the process of designing computational epitope-based vaccines, outlining the necessary steps, resources, databases, software, and all other requirements for researchers to design a multi-epitope vaccine comprehensively. This process involves identifying potent antigenic protein sequences, predicting epitopes, designing the vaccine, and validating the designed vaccine. analyzes the entire field of epitope-based vaccine design, attempting to describe a complete guide for each stage of multi-epitope vaccine design based on bioinformatics tools.

This section examines the key challenges, strengths, and weaknesses of current bioinformatics approaches and shows ways to optimize multi-epitope structures, it also gives researchers a complete picture of proven methods and new technologies, helping them make smart choices in vaccine development projects.

### 4.1 Current approaches in Vaccine design using bioinformatics tools

As mentioned in the article, one of the new approaches in the design of vaccines based on bioinformatics is to use the genome of pathogens to identify candidate epitopes or so-called reverse vaccinology. Vaccine design with the reverse vaccinology approach is a new model of vaccine design that has been developed with the advancement and integration of bioinformatics.

Before this approach, the emergence of other models, such as epitope-based design in the early 2000s as an alternative to traditional vaccines, was a tremendous advance in this science. This approach focuses on the identification and use of specific T-cell and B-cell epitopes to generate targeted immune responses. Tools like IEDB [125] (Immune Epitope Database) and NetMHC [137] began providing platforms for epitope prediction, helping researchers identify immunogenic peptides based on their binding affinity to Major Histocompatibility Complex (MHC) molecules. These tools allowed for the first time the rational design of vaccines based on computational predictions rather than empirical methods.

After that, more advanced approaches emerged, such as protein structure prediction, which helped to better understand epitopes, and immune-informatics, which helped scientists predict the immune response of different types of antigens and their corresponding epitopes.

protein structure prediction tools like I-TASSER, Rosetta, and AlphaFold [230] could predict the three-dimensional structure of antigens, which was crucial for the design of subunit and multi-epitope vaccines and could identify epitopes more accurately, allowing for better-targeted vaccines.

Immuno-informatics was the result of the intersection of immunology and bioinformatics, using machine learning, statistical models, and data Mining to predict the immune response to various antigens and their epitopes. These tools allowed researchers to predict not just the location of epitopes, but also their ability to trigger immune responses, marking a significant step towards designing more effective vaccines. VaxiJen [86] (for antigenicity prediction) and IEDB Analysis Resource were widely used for predicting the immunogenicity of peptides. ProtScale and EpiJen helped in analyzing antigenic properties based on sequence data.

After the development of immune-informatics, reverse vaccinology emerged. By examining the genomes of pathogens to find potential epitopes without relying on traditional methods like in vivo testing, took an essential step in vaccine design, especially for pathogens whose biology was poorly understood [231]. With the completion of genome sequencing projects, bioinformatic tools like GenBank, BLAST, and OrthoMCL [232] became integral in identifying antigenic proteins for vaccine development.

The next step in vaccine design was the introduction of multi-epitope vaccines. These vaccines, which incorporate several different epitopes from different antigens, have been shown to elicit a stronger immune response and produce higher quality immune cells.

Multi-epitope vaccine design is heavily reliant on epitope prediction tools (e.g., IEDB, BepiPred) and molecular docking simulations (e.g., AutoDock, HADDOCK) to predict interactions between the epitopes and the immune system.

Multi-epitope vaccines improve vaccine efficacy by targeting multiple immune pathways, making them particularly useful for complex diseases like cancer, HIV, and influenza.

Another paradigm occurred in 2015 with the advent of artificial intelligence and machine learning-based algorithms, opening up a new frontier in vaccine design and exciting possibilities for the future. This integration allows analysis of vast datasets to predict antigenicity, vaccine efficacy, and potential side effects [233].

AI-based tools can handle complex datasets, making them capable of designing vaccines in silico with high precision. These tools also enhance the ability to predict how the immune system will respond to different vaccine candidates, accelerating the process of vaccine discovery [234].

Notable tools include DeepImmuno [235], Vaxign [236] (an AI-based vaccine design tool), and AlphaFold [230], which has significantly improved the prediction of protein folding and epitope recognition, and The EpiScan [237] framework marks a major breakthrough in antibody-specific epitope prediction and shows better performance than existing methods.

Systems Biology Approaches like NGS technologies and multi-omics (such as genomics, proteomics, and metabolomics) have been integrated into vaccine design. Their integration has opened up new possibilities, enabling the identification of novel antigens and understanding immune responses at a molecular level.

These approaches not only allow for identifying novel vaccine targets and biomarkers for vaccine efficacy, but also provide practical solutions for understanding vaccine-induced immunity and its variability across individuals.

NGS platforms, such as Illumina [238] and PacBio [239], combined with bioinformatics pipelines, are now commonly used to sequence pathogen genomes or host immune responses. Their widespread use provides a reliable and comprehensive view of the immune system's response to pathogens and vaccines.

Computational modeling and simulation of the immune system is another approach used in recent years to design vaccines. Advanced computational models are being employed to simulate the immune response to vaccine candidates, allowing researchers to predict how vaccines will interact with immune cells and tissues. These simulations help to optimize vaccine formulations and assess potential immune responses in silico. These tools reduce the need for large-scale animal testing by providing accurate predictions of vaccine performance, speeding up the design and optimization phases.

Platforms like ImmSim [240], Simimmune, and GROMACS [241] (for molecular dynamics simulations) are used to model immune cell interactions and vaccine efficacy.

These new approaches, from epitope-based vaccine design to Computational modeling and AI-powered vaccine prediction, reflect the growing complexity of bioinformatics tools in vaccine development. By leveraging computational power and advanced biological understanding, these methods enable more accurate, faster, and cost-effective vaccine design, especially in response to emerging diseases like COVID-19, Zika, Ebola, and malaria, as well as chronic diseases like cancer.

Each of these approaches, in addition to its strengths and advances in the science of vaccine design, also has disadvantages and weaknesses. It is important to address and study both aspects to provide a balanced and objective analysis. This will reassure readers about the fairness of the assessment. An important example and review of these steps can be found in the article by Zikun Yang and his colleagues [242], who developed DeepVacPred and effectively implemented these steps in the best possible way.

In Table 13, a comprehensive review of the strengths and weaknesses of these approaches is presented. This thorough review will provide the audience with a confident understanding of the various vaccine design approaches.

**Table 13:** Comparison of various computational approaches used in vaccine design, highlighting their strengths and weaknesses.

Approach	Strengths	Weaknesses
Immuno-informatics	Cost-effective, rapid, highly specific epitope selection	Prediction limitations, lacks full immunological complexity
Reverse Vaccinology	Efficient, scalable, targets conserved epitopes, Broad Pathogen Coverage, High-Throughput Capability	depends on genome data, Reliance on Prediction Algorithms, Limited Insight on Epitope Functionality
Structural Bioinformatics	Accurate in binding affinity prediction, rational design support	Computationally intensive, limited structural data availability
Systems Biology	Comprehensive insight, potential for personalized vaccines	Data/model complexity, costly resources
AI/ML-Based Approaches	High precision, adaptive, pattern recognition	Data dependency, complexity, interpretability issues

### 4.2 key challenge in multi-epitope vaccine design

Another important issue that should be addressed in the discussion after comparing the approaches is the key challenges in vaccine design and the proposed solutions for them. The key challenges of multi-epitope vaccines are as follows.

#### 4.2.1 Accuracy of Epitope prediction

The accuracy of epitope prediction is vital for multi-epitope vaccine design but often faces challenges. False positives and false negatives can significantly affect vaccine effectiveness. These problems derive from dataset limitations and our incomplete knowledge of peptide-MHC interactions, which may result in computationally predicted epitopes failing to trigger the desired immune responses in practice [243].

One of the solutions for this challenge is using artificial intelligence and machine learning to increase the accuracy of predictions. Advances in artificial intelligence and machine learning (ML) have led to developing more sophisticated epitope prediction tools. ML models can better capture the complex features that determine binding affinity and immunogenicity by training on larger datasets that include more diverse peptide-MHC interactions. Deep learning models, such as convolutional neural networks (CNN) and recurrent neural networks (RNN), have shown improved prediction accuracy for MHC-I and MHC-II binding associations. These methods reduce false positives and false negatives, leading to more reliable epitope predictions [244].

#### 4.2.2 Genetic variability and HLA diversity

Human leukocyte antigen (HLA) polymorphisms vary significantly among populations, meaning an epitope effective in one genetic background may not work as well in another. Designing multi-epitope vaccines for broad population coverage is challenging due to the high variability in HLA alleles across individuals and ethnic groups [245]. This diversity complicates epitope selection, as an epitope may bind strongly to one HLA allele but weakly to another [246]. One solution for this challenge is HLA Supertype-Based Selection and Population-Specific Databases. To address the issue of HLA diversity, epitopes can be selected based on HLA supertypes, which are groups of HLA alleles with similar binding characteristics [246]. This approach allows vaccine designers to cover broader populations without including excessive epitopes. Additionally, population-specific databases are being developed to identify the most common HLA alleles within various ethnic groups, enabling more targeted epitope selection for different regions.

#### 4.2.3 Structural stability of multi-epitope constructs

Multi-epitope constructs, which consist of several peptides, can encounter stability challenges. Linking these peptides may cause conformational changes that impair immunogenicity or compromise stability. Maintaining the structural integrity of the vaccine construct is crucial for its stability, shelf life, and effectiveness. Bioinformatics tools used for modeling these constructs must ensure the stability of peptide bonds and achieve the correct conformation for optimal immunogenicity.

One solution for this challenge is Advanced Molecular Dynamics for Structural Refinement. Structural modeling and molecular dynamics (MD) simulations help optimize the stability of multi-epitope vaccine constructs. MD simulations allow researchers to observe peptide conformations and interactions under dynamic conditions, helping ensure that the construct maintains its stability and immunogenicity in a realistic environment. This approach helps identify optimal linker sequences and structural configurations, improving the overall stability and effectiveness of the vaccine [247].

#### 4.2.4 Prediction of immunogenicity and safety

Not all epitopes are safe or effective in vaccines. Some may trigger unwanted immune responses, such as cross-reactivity with human proteins, potentially causing autoimmune reactions. Predicting immunogenicity requires evaluating whether the immune system will respond to an epitope safely and in a controlled manner [248]. This safety prediction is complex due to gaps in understanding immune tolerance and cross-reactivity.

One solution for this challenge is cross-activity databases and Tolerance Prediction Algorithms. Cross-reactivity databases and tolerance prediction algorithms help filter out epitopes that could trigger autoimmune reactions. By cross-referencing epitopes with the human proteome, bioinformatics tools can reduce the likelihood of selecting epitopes similar to human proteins. Tolerance prediction algorithms assess the likelihood of epitopes being recognized as self-antigens, which is particularly important for preventing autoimmunity [249].

#### 4.2.5 Lack of standardization across tools

Various tools and algorithms exist for epitope prediction, each with methodologies and metrics. This lack of standardization leads to inconsistent results across studies, making validating and comparing findings difficult. Without standardized benchmarks, results from different tools may not be comparable, complicating efforts to make multi-epitope vaccine design reproducible and reliable [250].

A viable solution to this challenge is Standardized Benchmarking and Cross-Tool Validation. Implementing standardized benchmarking across epitope prediction tools would improve consistency and reliability in epitope selection. Cross-tool validation employs multiple prediction algorithms to create consensus predictions, thus reducing variability and boosting confidence in selected epitopes [251]. This strategy also promotes the creation of universal benchmarks, making results from different tools comparable and aiding vaccine developers in making more informed decisions.

#### 4.2.6 Translational gap between in-silico predictions and in-vivo efficacy

Bioinformatics tools often generate promising in-silico predictions that may not translate into effective in-vivo or clinical outcomes. This discrepancy can stem from differences in immune complexity, the limitations of computational models, or unexpected interactions in the human body that are not accounted for in silico. Bridging this translational gap ensures that computationally designed vaccines achieve real-world effectiveness [252].

One solution for this challenge is Experimental Validation Loops for Translational Improvement. Iterative validation loops involve refining computational predictions through experimental validation, such as in vitro assays, preclinical animal models, or human organoids. This approach allows for iterative adjustments based on experimental feedback, improving the translational reliability of in-silico predictions [253]. It also bridges the gap between computational models and real-world immune responses, providing a more straightforward path to clinical trials.

### 4.3 Future directions and recommendations

The rapid advancements in bioinformatics tools and computational biology have revolutionized multi-epitope vaccine design. However, the field is still evolving, and addressing the current challenges can significantly enhance the efficiency and applicability of these vaccines. This section outlines vital future directions and recommendations to guide research and development.

#### 4.3.1 Integration of artificial intelligence and machine learning

Machine learning (ML) and artificial intelligence (AI) offer transformative potential in vaccine design. Researchers can analyze massive datasets by integrating these technologies to identify patterns and improve epitope prediction accuracy. ML models trained on immunogenicity data could optimize epitope selection and predict immune responses in diverse populations. Further development of explainable AI models is crucial to ensure transparency and reproducibility in vaccine design.

#### 4.3.2 Development of comprehensive and real-time databases

Current epitope prediction tools rely heavily on pre-existing datasets, many of which are static and outdated. The creation of dynamic, real-time databases that incorporate data on new pathogens, variants, and immune responses is essential. These databases should integrate genomic, proteomic, and structural data from diverse geographic regions, ensuring better global population coverage.

#### 4.3.3 Multi-omics integration

Integrating multi-omics data—such as genomics, transcriptomics, and proteomics—can provide a holistic view of host-pathogen interactions. Systems-level analyses could enhance the identification of immunodominant epitopes and guide the design of more effective vaccines. Moreover, integrating host-microbiome data could provide insights into how gut flora modulates vaccine efficacy and immunity.

#### 4.3.4 Personalized vaccine design

Personalized vaccines tailored to individual genetic and immunological profiles represent the next frontier in immunotherapy. Advances in bioinformatics can facilitate the identification of epitopes most suitable for specific populations based on their human leukocyte antigen (HLA) types. This is particularly relevant for vaccines targeting diseases like cancer, where personalized neoantigen-based strategies are gaining traction.

#### 4.3.5 Enhancing structural biology techniques

The inclusion of structural bioinformatics in vaccine design has shown promise in identifying epitopes with high binding affinities to immune receptors. Future efforts should focus on improving computational modeling of antigen-antibody and antigen-T-cell receptor interactions. Tools that simulate antigen processing and epitope presentation more accurately will enhance the precision of vaccine design.

#### 4.3.6 Addressing pathogen Evolution

Emerging and re-emerging pathogens frequently undergo genetic mutations that render vaccines less effective. Predictive models must be developed to anticipate escape mutations and design epitope-based vaccines targeting conserved and critical regions of the pathogen. This approach can be beneficial for fast-evolving viruses like influenza and SARS-CoV-2.

#### 4.3.7 Experimental validation and bridging the in silico-in vivo gap

While computational methods have accelerated vaccine design, there is a critical need for robust experimental validation. Collaborative efforts between computational biologists and immunologists should focus on creating streamlined workflows that bridge in silico predictions with in vitro and in vivo testing. Developing high-throughput experimental platforms can validate epitopes more efficiently and ensure the clinical applicability of vaccine candidates.

#### 4.3.8 Democratizing bioinformatics tools

Bioinformatics tools should be open-source, user-friendly, and adaptable to diverse research settings to ensure equitable access to advanced vaccine technologies. Training programs and workshops can also empower researchers in low-resource settings, enabling them to leverage these tools effectively.

## 5 Conclusion

This article aims to comprehensively identify all steps effective in the process of epitope-based vaccine design. First, we introduce essential matters in the immune system's introduction, then clarify all steps needed to design this type of vaccine. The important thing in this regard is that we introduce the most important, famous, and well-known software, servers, and resources that scientists can use at each step. The method of an epitope-based vaccine is a powerful combination of scientific knowledge and practical application in immunization. These vaccines utilize specific immune-activating elements in pathogens to develop tailored solutions for various diseases. As demonstrated by successful preclinical and clinical trial results, interdisciplinary collaboration, including immunology, bioinformatics, structural biology, and clinical research, has made novel concepts globally meaningful. Improving the prediction of epitopes, exploring personalized vaccines, and overcoming complexity are the future of this field. Even though there are difficulties, designing vaccines using epitopes is a hopeful sign in the ongoing battle against diseases.

## 6 Ethics Statement

This study did not involve human participants, human data, or human tissues, nor did it include any experimentation on animals. As such, no ethical approval was required. All analyses were performed on publicly available datasets, and no new data collection or experimentation was conducted. The research complies with all relevant laws and institutional guidelines.

## CRediT authorship contribution statement

**Esmaeil Roohparvar Basmenj:** Conceptualization, Data curation, Formal analysis, Investigation, Methodology, Project administration, Resources, Software, Supervision, Validation, Visualization, Writing – original draft, Writing – review & editing. **Susan Radman Pajhouh:** Data curation, Formal analysis, Investigation, Validation, Writing – original draft, Writing – review & editing. **Afsane Ebrahimi Fallah:** Formal analysis, Methodology, Validation, Visualization. **Rafe naijian:** Conceptualization, Formal analysis, Software, Validation, Visualization. **Elmira Rahimi:** Data curation, Formal analysis, Methodology. **Hossein Atighy:** Methodology, Resources, Software. **Shadan Ghiabi:** Investigation, Methodology. **Shamim Ghiabi:** Investigation, Methodology.

## Declaration of competing interest

The authors declare that they have no known competing financial interests or personal relationships that could have appeared to influence the work reported in this paper.
